# Optimization of Ultrasound-Assisted Extraction of *Verbascum sinaiticum* Leaves: Maximal Phenolic Yield and Antioxidant Capacity

**DOI:** 10.3390/foods13081255

**Published:** 2024-04-19

**Authors:** Alemu Belay Legesse, Shimelis Admassu Emire, Minbale Gashu Tadesse, Debebe Worku Dadi, Shimelis Kebede Kassa, Timilehin Martins Oyinloye, Won Byong Yoon

**Affiliations:** 1 Department of Food Science and Biotechnology, College of Agriculture and Life Sciences, Kangwon National University, Chuncheon 24341, Republic of Korea; alemu.belay@aait.edu.et (A.B.L.); oyinloyetm@kangwon.ac.kr (T.M.O.); 2 Elder-Friendly Research Center, Agriculture and Life Science Research Institute, Kangwon National University, Chuncheon 24341, Republic of Korea; 3 School of Chemical and Bio Engineering, Addis Ababa Institute of Technology, Addis Ababa University, Addis Ababa P.O. Box 385, Ethiopia; shimelis.admassu@aait.edu.et (S.A.E.); shimelis.kebede@aait.edu.et (S.K.K.); 4 Department of Food Engineering, College of Engineering, Debre Berhan University, Debre Berhan P.O. Box 445, Ethiopia; 5 Department of Chemistry, Natural and Computational Sciences, Debre Berhan University, Debre Berhan P.O. Box 445, Ethiopia; minbalegashu2012@gmail.com; 6 Department of Food Process Engineering and Postharvest Technology, Institute of Technology, Ambo University, Ambo 2040, Ethiopia; debebeworku2010@gmail.com

**Keywords:** *Verbascum sinaiticum*, ultrasound extraction, antioxidant, optimization, UHPLC-ESI-Q-TOF-MS/MS, metabolite

## Abstract

*Verbascum sinaiticum* (*Qetetina or yeahya Joro*) is a medicinal plant with secondary metabolites such as phenolics, flavonoids, glycosides, saponins, and alkaloids. This study was designed to optimize the ultrasonic-assisted extraction (UAE) parameters to enhance the phenolic content and characterize the phenolic compounds using ultra-high-performance liquid chromatography, coupled with electrospray ionization quadrupole time-of-flight tandem mass spectrometry (UHPLC-ESI-QTOF-MS/MS), and antioxidant activities in *Verbascum sinaiticum* extract. Extraction time, sample-to-solvent ratio, and extraction temperature were considered for UAE optimization. It was found that UAE generated the highest extraction yield (21.6%), total phenolic content (179.8 GAE mg/g), total flavonoid content (64.49 CE mg/g), DPPH (61.85 µg/mL), and ABTS (38.89 µg/mL) when compared to maceration extraction. Metabolite analysis in this study showed the detection of 17 phenolic compounds, confirming antioxidant capacities. The optimization parameters have significant effects on phenolic compounds. Scanning electron microscopy showed the presence of structural changes when UAE was used over the maceration method. The optimized UAE parameters for extraction temperature (41.43 °C), sample-to-solvent ratio (36.32 g/mL), and extraction time (33.22 min) for TPC were obtained. This study shows the potential application for UAE of *Verbascum sinaiticum* leaves in the development of pharmaceutical and nutraceutical products.

## 1. Introduction

*Verbascum sinaiticum* (*V. sinaiticum*) is a medicinal plant belonging to the Scrophulariaceae family. Extensive ethnomedicinal activities of *V. sinaiticum*, utilizing its aerial parts, stem, flowers, roots, and leaves, have been reported, including antioxidant, antibacterial, antihyperlipidemic, anticancer, antiviral, cytotoxic, and wound-healing activities [1,2,3]. Phytochemical analysis of *V. sinaiticum* leaves has revealed the presence of flavonoids and phenolic compounds, including verbascoside, apigenin-7-glucoside, arenariosides, cistanosides, caffeic acid, chlorogenic acid, quercetin, myricetin, and kaempferol [4]. Additionally, flavonolignans and flavones have been isolated from the aerial parts of *V. sinaiticum* [4,5,6,7,8,9,10]. Flavones such as luteolin, luteolin-7-glucoside, acacetin-7-galactoside, and chrysoeriol-7-glucoside, flavonolignans like hydrocarpin and sinaiticin, mullein saponins, and phenolic glycosides are also obtained from the aerial part of the plant [11]. Furthermore, methanolic extracts from *V. sinaiticum*, as well as extracts from other plant species, demonstrate broad-spectrum antibacterial activity [1,12]. *V. sinaiticum* is recognized for its positive pharmacological effects on organisms, attributed to the presence of metabolites such as flavonoids, phenolics, glycosides, saponins, anthraquinones, and alkaloids [4,13].

In recent years, the pharmaceutical, cosmetic, and food industries have begun adopting new extraction techniques such as ultrasound-assisted extraction (UAE) to address sustainability concerns associated with conventional methods. The application of ultrasound has emerged as a promising method for extracting oil from plants. Alongside the extraction method, numerous technological parameters influence the extraction yield in the industrial process, including solvent composition, solid-to-solvent ratio, particle size, extraction temperature, frequency, power, pressure, extraction time, pH, and solvent type [14]. These new techniques aim to reduce energy consumption, time, emissions, and costs while enhancing product safety and quality [9,14,15]. UAE has been utilized to extract bioactive compounds from plants, algae, fruits, bacteria, fungi, and animals [14,15,16]. Its applicability in both laboratory and industrial settings indicates its versatility and widespread adoption across different scales of operation. This rapid extraction method is attributed to various ultrasound effects that lead to cell wall disruption, improving mass transfer, and reducing solvent consumption [15,16,17,18,19,20]. Furthermore, UAE is crucial for extracting phenolic compounds from samples to produce high-quality, target-rich chemical extracts in shorter extraction durations with minimal to no usage of organic solvents [21,22,23,24].

One of the most promising methodologies for plant metabolic profiling involves employing ultra-high-performance liquid chromatography, coupled with electrospray ionization quadrupole time-of-flight tandem mass spectrometry (UHPLC-ESI-QTOF-MS/MS). Compared to classical liquid chromatography, this technique offers improved separation, faster analyses, and heightened sensitivity [25]. UHPLC-ESI-QTOF-MS/MS is a highly potent separation method extensively utilized for purifying, identifying, or quantifying one or several components simultaneously in mixtures within the pharmaceutical industry, biological sciences, and chemical research. The integration of chromatographic separation with tandem mass spectrometry allows for the acquisition of robust data with rapid acquisition rates and high mass accuracy across a broad mass range. This aids in the quantitative and qualitative analysis of molecules within complex matrices by minimizing interference from the matrix. However, there were no reports of the physicochemical, functional, structural, metabolite, and phenolic yield, together with the antioxidant capacity of *V. sinaiticum* UAE extract. *V. sinaiticum* abundant sources of health-promoting bioactive compounds, including phenolic and polyphenolic compounds, tannins, saponins, and terpenoids, contribute to the plant’s therapeutic properties and antimicrobial effects [1,2,3,13]. Therefore, UAE could potentially improve the extraction of polyphenols from *V. sinaiticum* and enhance the antioxidant capacity of the polyphenol-rich extract. Notably, the efficacy of UAE is contingent upon various factors, including sonication time, solvent-to-solute ratio, and extraction temperature. Therefore, the objective of this study was to optimize the UAE parameters to enhance the phenolic content by applying central composite design (CCD) methods in order to (a) extract high phenolic content, (b) integrate antioxidant capacity with their phenolic compounds’ full scan characterized by UHPLC-Q-TOF-MS/MS and FTIR in *V. sinaiticum*, and (c) evaluate the effects of extraction conditions on antioxidant capacity and total phenolic content. These findings served as foundational data for potential industrial production for antioxidant, antibacterial, anticancer, and pharmaceutical applications and the continued exploration of *V. sinaiticum* as a source of phenolic, flavonoid, and other antioxidant compounds [25,26].

## 2. Material and Methods

### 2.1. Raw Material

Fresh *V. sinaiticum* leaves were collected from the Bella district, with coordinates of 9.005401° and longitude coordinates of 38.763611°, in Addis Ababa, Ethiopia. The collection of plant material was authorized by Botanist Dr. Feleke Woldeyes at the Ethiopian Biodiversity Institute in Addis Ababa, Ethiopia. A voucher specimen, 182/2837/2014 EC (EBY-182), was provided and deposited for future reference. The leaf samples were physically cleaned and washed with tap water. Then, the sample was shade-dried (22–27 °C) for 5 days. Ultimately, the dried leaves were pulverized using a miller (Dietz-Motoren KG, Retsch Gmbh, Haan, Germany) and sieved through a 20-mesh filter. Finally, they were packed in an airtight plastic bag and stored away from light until analysis.

### 2.2. Chemicals and Reagents

All chemicals and reagents were of analytical grade. Ethanol, Folin–Ciocâlteu reagent, water (HPLC grade), ethyl alcohol, acetonitrile (HPLC grade), sodium nitrite, sodium carbonate, ABTS (2,2′-azinobis-3-ethylbenzothiazoline-6-sulphonic acid diammonium salt), aluminum chloride, catechin (2,2-diphenyl-1-picrylhydrazyl: DPPH), gallic acid, and sodium hydroxide were purchased from Sigma–Aldrich (Seoul, Republic of Korea). Deionized water was obtained from Kangwon National University, Republic of Korea.

### 2.3. Extraction Method

#### 2.3.1. Maceration Extraction

Maceration extraction (ME) of *V. sinaiticum* was conducted according to [27,28]. *V. sinaiticum* powder was mixed with 70% (*v*/*v*) ethanol in a ratio of 1:30 g/mL. It was then placed in a shaking stomacher (JSSB-50T; JS Research Inc., Gongju-si, Republic of Korea) at 170 rpm and 25 °C for 72 h (h). Subsequently, the crude extracts were centrifuged at 3250× *g* for 7 min (min), followed by filtration through Whatman No. 1 filter paper. The filtrate was then dried using a rotary evaporator (N-1001; EYLA, Tokyo, Japan) under vacuum conditions at 40 °C. Further drying was carried out using a freeze dryer for 3 days, and the resulting extract was stored in a −80 °C deep freezer until further analysis [29]. The extract yield was computed as:

(1) $$\mathrm{Extract}\;\mathrm{yield}\;(\%)=\;\frac{Extract\;weight}{Initial\;sample\;weight}\times 100$$

#### 2.3.2. Ultrasound-Assisted Extraction (UAE)

The ultrasonic processor utilized in this study consisted of an ultrasonic probe with a power of 750 W and a frequency of 20 kHz (VCX 750, Sonics and Materials Inc., Newtown, CT, USA). This experimental design was based on the results of preliminary experiments, where the ultrasound extraction time, temperature, and solvent-to-solute ratio were the variable values, while the 40% amplitude was the only fixed variable. The UAE was conducted following the method of Babamoradi N et al. [19], with minor adjustments. The sample was placed in 70% ethanol, and then the leaves were suspended in ethanol and ultrasonicated under the parameters shown in Table 1. The sonicated solutions were immediately cooled to 25 ± 1 °C using ice cubes and then centrifuged at 4500× *g* for 5 min. The supernatant was collected and concentrated using a rotary evaporator (N-1001; EYLA, Tokyo, Japan). Finally, the crude extract was dried by freeze-drying and stored at −80 °C.

#### 2.3.3. Response Surface Methodology and Optimization of UAE

To optimize conditions in the UAE, independent factors such as sonication time, solvent-to-solute ratio, and extraction temperature were tested in 20 experimental runs to determine the optimal conditions for total phenolic content (TPC) extraction from *V. sinaiticum* (Table 1). For response variables, a second-order polynomial equation was determined as:

(2) $$Y_{n}=\beta_{0}+\beta_{1}x_{1}+\beta_{2}x_{2}+\beta_{3}x_{3}+\beta_{11}{x_{1}}^{2}+\beta_{22}{x_{2}}^{2}+\beta_{33}{x_{3}}^{2}+\beta_{12}{x_{2}}^{2}+\beta_{13}{x_{3}}^{2}+\beta_{23}{x_{3}}^{2}$$

where Y_n_ is TPC, variables β_0_, β_i_, β_ii_ and β_ij_ are intercept, linear regression coefficient for i^th^ factor, quadric, and interaction effect term. X_i_ and X_j_ are coded the independent variables. k is the number of tested variables.

#### 2.3.4. Total Polyphenol Content

TPC was determined calorimetrically using the Folin–Ciocâlteu technique [30]. The solution consisted of 0.2 mL of the extract mixed with 2.5 mL of 10% Folin–Ciocâlteu reagent (FCR). Then, 2 mL of 7.5% sodium carbonate solution with a concentration of 75 g/mL was added. The sample was heated to 50 °C for 10 min and allowed to cool. The absorbance was measured at 750 nm using a Spectra i3x plate reader (Molecular Devices, LLC., Seoul, Republic of Korea). A calibration curve was established using gallic acid standard, and the results were expressed in mg GAE/g dw.

#### 2.3.5. Total Flavonoids Content

TFC was evaluated using the method of Zhishen et al. [31]. Initially, 0.5 mL of the extract was combined with 2.5 mL of distilled water and 0.15 mL of 5% sodium nitrite. The mixture was allowed to stand for 6 min, after which 0.3 mL of aluminum chloride (10% m/V) was added and thoroughly mixed. Following this, 1 mL of 1.0 M sodium hydroxide was added, followed by 0.55 mL of distilled water. The resulting mixture was vortexed and left to stand for 15 min. Finally, the concentration was measured at 510 nm using a UV-Vis spectrophotometer (Optizen 2120UV; Mecasys Co., Ltd., Daejeon, Republic of Korea). A calibration curve for catechin was established for quantification, and the outcomes were expressed as (mg CE/g DW) dry extract of the sample.

### 2.4. Antioxidant Capacity of V. sinaiticum Leaf Extract

#### 2.4.1. DPPH Radical Scavenger

The radical scavenging activity of the *V. sinaiticum* leaf extract was measured using 1,1-diphenyl-2-picrylhydrazyl (DPPH) [32], with a slight modification. Three milliliters of DPPH solution (0.004%) were added to the extract, standard, or blank solution (1 mL). The mixture was incubated in darkness at room temperature for 30 min. The absorbance was measured against the blank using a spectrophotometer at 517 nm (Molecular Devices LLC., Spectra i3x Gangnam-gu, Seoul, Republic of Korea) [33]. Then, the data were expressed as IC_50_.

#### 2.4.2. ABTS^•+^ Radical Scavenging Test

The ABTS assay was conducted as per the previous procedure [33,34]. 0.9 mL of the ABTS^•+^ solution was combined with 0.1 mL of the extract solution, and the mixture was incubated at 30 °C for 30 min. The absorbance at a wavelength of 734 nm was recorded. The ABTS scavenging percentage was expressed as the IC50.

### 2.5. Phytochemical Profiling by UHPLC-ESI-QTOF-MS/MS

Plant extracts were analyzed by liquid chromatography on an Agilent 1290 series LC system using a YMC-Pack Pro C18 column (150 × 4.6 mm I.D., 3 µm, 12 nm) at 40 °C. The LC conditions were as follows: flow rate, 0.5 mL/min; solvent A, 0.1% formic acid in DW; solvent B, acetonitrile (ACN). The gradient was from 10% to 100% B over 35 min, kept for 5 min, and then returned to 10% for 10 min. Five microliters of each sample were analyzed by electrospray ionization in positive and negative mode using an Agilent 6545 quadrupole time of flight mass spectrometry. Mass spectral data were obtained within the *m*/*z* range of 100–1000 amu. The source parameters were configured as follows: a drying gas temperature of 320 °C, a drying gas flow rate of 8 mL/min, and a nebulizer pressure of 35 psi. Features were compared to reported compounds from the study plant and in the Metlin database. Based on spectral similarities with fragments predicted online databases such as Metlin, and available data in the literature, putative assignments were acquired for smooth baseline, and identifications were adopted when no database or literature was found [10,25,26].

### 2.6. ATR-FTIR

The powdered extract sample of *V. sinaiticum* was placed on the diamond crystal surface of the attenuated total reflection (ATR) cell of the FTIR spectrometer (model: iS50, Thermo Scientific, Waltham, MA, USA). The FTIR analysis covered wave numbers from 400 cm^−1^ to 4000 cm^−1^, with an average scanning rate of 1 cm^−1^ resolution [29].

### 2.7. Scanning Electron Microscopy (SEM) Analysis

The extract residues were dried at 40 °C for 4 h. The dried residues were coated with gold under vacuum conditions, then examined using an accelerated voltage of 15 kV under high vacuum conditions with a magnification of to 500X. The morphological analysis was done using SEM (JSM-7500F; JEOL Ltd., Tokyo, Japan) [35].

### 2.8. X-ray Diffraction (XRD) Analysis

The dried leaves were analyzed using a diffractometer (XRD6000, Shimadzu, Kyoto, Japan) to examine crystalline structure. Radiation with a wavelength of 0.154 nm was produced by filtering monochromatic light at 40 kV and 40 mA. The sample was scanned at room temperature within the 2θ range of 5–40°, with 0.04° intervals and a scanning speed of 2° per min.

### 2.9. Statistical Analysis

Statistical analysis was conducted utilizing Design Expert 13 software (Stat-Ease, Inc., Minneapolis, MN, USA). Analysis of variance (ANOVA) and coefficients of determination (R^2^), coupled with Tukey’s test *p* ≤ 0.05, were used to evaluate the regression model’s goodness of fit. Three-dimensional response surface methodology (RSM) analyses were carried out to determine the optimal extraction conditions.

## 3. Result and Discussion

### 3.1. Effects of Ultrasonic-Assisted Extraction Parameters on Extraction Yield, Bioactive Compounds and Antioxidant Capacity

The mean extraction yield values are presented in Table 2. The highest yield of 21.60% was obtained with an extraction time of 30 min, a solvent-to-solute ratio of 30 mL/g, and an extraction temperature of 40 °C. Conversely, the lowest extraction yield of 19.35% was obtained with an extraction time of 13.2 min, an extraction temperature of 40 °C, and a solvent-to-solute ratio of 40 mL/g. The ANOVA analysis of UAE indicates a positive effect of linear factors such as extraction temperature (X_1_), ranging from 30 to 50 °C (*p* > 0.05), which positively impacts TPC extraction, peaking at 40 °C, after which a slight decline is observed. This increase is attributed to enhanced polyphenol solubility in deep eutectic solvents with rising X_1_, facilitating mass transfer from *V. sinaiticum* cells. However, at higher temperatures, some heat-sensitive TPC may decompose, leading to a slight decrease in TPC at 50 °C. Thus, X_1_ of 30 °C, 40 °C, and 50 °C were chosen for subsequent experiments [17,36,37,38]. Extraction times (X_2_), ranging from 20 to 40 min (*p* < 0.0005), can indeed impact the TPC of extracts. Initially, the TPC of the extracts increased as X_2_ progressed from 20 to 30 min, but then it decreased as the X_2_ exceeded 30 min. During the early stage of extraction, the intracellular polyphenols encountered minimal diffusion resistance, attributed to the highly effective ultrasound-induced damage to cell structures within the sample. However, with further prolongation of the extraction process, polyphenol solubility reaches its peak. At this point, some of the TPC may begin to decompose due to the associated high temperatures and lengthy extraction times [17,29,36]. Solvent-to-solute ratios (X_3_), ranging from 20 to 40 mL/g (*p* < 0.0005), also play a role in affecting the TPC. When X_3_ increased from 1:20 to 1:30 g/mL, the increase can be attributed to the enlarged contact area between the solute and solvent, resulting in enhanced diffusion of TPC from the intracellular sample matrix into the solvent. However, when X_3_ was further elevated from 1:30 g/mL to 1:40 g/mL, a very small decline (*p* > 0.05) in TPC was observed [24,38]. There is also a positive effect of the interaction between X_1_X_2_ (*p* > 0.05), and negative effects are observed for interaction X_2_X_3_ (*p* < 0.005) and X_1_X_3_ (*p* > 0.05) [36,39]. For the extraction of bioactive compounds utilizing *Allium sativum* leaves by Shekahar S et al. [36] and natural pigment from annatto seeds by Yolemeh M [39], a similar pattern was reported.

The highest TPC value, 179.8 mg GAE/g, was obtained at X_1_ of 40 °C, X_2_ of 30 min, and X_3_ of 30 mL/g, which aligns with a similar result reported previously [29]. The UAE interaction term of X_1_X_2_ (*p* < 0.05) had a significant positive impact on the extraction of TPC. At a higher X_1_, the yield of TPC improves. The X_1_, X_2_, and X_3_ factors had an impact on the antioxidant components of *V. sinaiticum* leaves extracted using UAE. A middle value of X1 at 40 °C, X_2_ at 40 min, and X_3_ at 30 mL/g resulted in the highest overall phenolic concentration. TPC values decreased when X_1_ was at 40 °C and X_2_ was at 40 min, a trend similar to that reported by Elnour A. et al. [37]. Similar results have been noted for the extraction of antioxidant components from blackberry leaves using UAE [37].

The highest TFC value of 64.49 mg CE/g was obtained with X_1_ of 40 °C, X_2_ of 30 min, and X_3_ of 30 mL/g. TFC is significantly and positively influenced by ultrasound X_1_ (*p* < 0.005), X_2_ (*p* < 0.001), and X_3_ (*p* < 0.001). However, there are negative effects from quadratic factors. TFC decreased in a non-significant (*p* > 0.05) way as X_1_ increased from 40 °C to 50 °C. The negative quadratic impact (*p* > 0.05) predominates with higher temperatures, resulting in an increase in TFC extraction. Similarly, a rise in TFC was observed in relation to X_2_ treatment from 20 to 30 min, which then declined noticeably as X_2_ increased to 40 min. The TFC content rises with increasing X_1_. This is because rising temperatures cause the solvent’s surface tension to decrease, while increasing vapor pressure causes cavitation bubbles to form at lower acoustic intensities, thus increasing TFC [14,37].

The DPPH and ABTS scavenging assays are commonly used to measure antioxidant capacity. The mean values of the experimental data and ANOVA analysis are shown in Table 2. The highest antioxidant values for ABTS and DPPH were 61.85 IC50 (µg/mL) and 38.89 IC50 (µg/mL), respectively, obtained at an X_1_ of 40 °C, X_2_ of 30 min, and X_3_ of 30 mL/g. In contrast, the lowest values were 44.93 IC50 (µg/mL) for DPPH at an X_1_ of 40 °C, an X_2_ of 30 min, and an X_3_ of 13.2 mL/g, and 28.24 IC50 (µg/mL) for ABTS at an X_2_ of 20 min, an X_1_ of 30 °C, and an X_3_ ratio of 20 mL/g. Antioxidant capacity (DPPH and ABTS) was shown to be positively affected by all linear and interactive terms, such as X_1_X_2_, while other interactive and quadratic terms had negative effects on the UAE. The surge in antioxidant capacity may be caused by cavitation, which increases the thermal effect, leading to the disruption of the plant cell structure. This disruption results in the release of antioxidants previously bound within the cell. Additionally, increased antioxidant capacity can also be attributed to a higher polyphenol content in *Allium sativum* leaf extract, which is caused by cavitation during UAE [17]. The decrease in antioxidant capacity is further due to the damaging effect of oxidation during prolonged UAE. A similar pattern of antioxidant capacity was reported in *Allium sativum* leaves [17].

### 3.2. Optimization of UAE for Phenolic Compounds from V. sinaiticum

According to the CCD of TPC values in *V. sinaiticum* leaf extract, 20 experimental runs were performed. The extraction conditions and the TPC of each experimental run are presented in Table 3. The UAE variables were optimized for TPC extraction using the CCD. For every response, the analysis of variance (ANOVA) revealed a significant (*p* < 0.05) model F-value with a non-significant lack of fit. There were fewer variations around the mean value and a good fit between the experimental data and the coefficient of determination (R^2^).

The entire quadratic model was demonstrated to be more suitable for the extraction of TPC models based on the values of R^2^-adj and R^2^. For the TPC content values, R^2^-adj and R^2^ were 89.35% and 79.77%, respectively. The TPC coefficient values and corresponding *p*-values are displayed in Table 3. Except for the extraction temperature, all linear terms were significant. The quadratic interaction terms of X_1_∗X_3_, X_2_∗X_3_, and X_2_∗X_3_ were found to be insignificant among the quadratic coefficients. On the other hand, quadratic terms of X_1_^2^, X_2_^2^, and X_3_^2^ were found to be significant. The predicted models for the TPC were computed using Equation (3):

(3) $$Y_{\mathrm{TPC}}=176.15+1.03X_{1}+4.15X_{2}+4.74X_{3}+1.22X_{1}*X_{2}-0.95X_{1}*X_{3}-1.1X_{2}*X_{3}-2.88X_{1}^{2}-5.62X_{2}^{2}-3.37X_{3}^{2}$$

where Y_TPC_ is the predicted responses (TPC), extraction temperature, $X_{1}$; extraction time, $X_{2};$ solvent-to-solute ratio, $X_{3}$.

ANOVA analysis of the quadratic polynomial model revealed significance. A high F-value and a low *p*-value for each term in the models would indicate greater significance on the corresponding response variable [39]. Therefore, the linear term of $X_{3}$ and the quadric term of $X_{2}^{2}$ had the largest effect on the extraction TPC. The two linear terms, namely $X_{2}$ and $X_{3}$, exhibited a significant effect (*p* < 0.05) on the TPC. Conversely, X_1_ did not display a significant effect (*p* > 0.05), whereas all quadratic terms showed a significant impact (*p* < 0.05) on the TPC.

**Figure 1:**
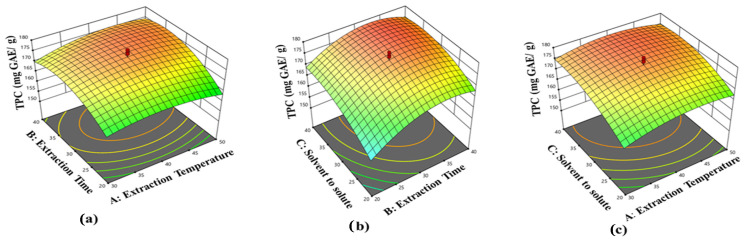
Response surface: (**a**) temperature and solvent-to-solute ratio; (**b**) sonication time and temperature; (**c**) extraction time and solvent-to-solute ratio on TPC of the *V. sinaiticum* extract.

Response surface plots were generated based on Equation (3) to ascertain the optimal conditions for the TPC of *V. sinaiticum* leaves extracted via UAE. The optimal conditions for *V. sinaiticum* extraction by UAE, suggested by the model to achieve high TPC, were specified as follows: a 40 min X_1_, a 40 °C X_2_, and a 1:30 g/mL X_3_ to reach the optimum yield of TPC values of 179.8 mg GAE/g. Figure 1 depicts the effects of the experimental levels of tested variables on the response. The plots came in a variety of forms, indicating various interactions between factors. Figure 1a shows the interaction effect of X_1_ and X_2_ and their influence on the TPC. The TPC of *V. sinaiticum* extract improved when X_2_ increased. Ultrasound waves require a certain time to stimulate cell wall interference and then release the extract. A similar effect of X_1_ on the TPC of *V. sinaiticum* extract was observed. Results showed that as X_1_ increased, the solubility of *V. sinaiticum* also increased, thereby improving the TPC. The TPC of *V. sinaiticum* extract also increased due to other reasons such as higher solvation, increasing material porosity, and mass transfer, as confirmed [9]. The impact of X_1_ on *V. sinaiticum* TPC and its phytochemical content was confirmed by a previous study conducted by Elnour et al. [29], which showed that increases in X_1_ led to increased TPC in the content of phenolic components of samples. However, the results showed that with an increase in X_1_ from 30 to 40 °C, the extraction TPC increased, and there were increases in TPC with increasing X_1_ due to the mass transfer produced by the increase in *V. sinaiticum* solubility and the decrease in solvent viscosity. The TPC was maximal at a temperature of 41.42 °C and decreased with further increases in temperature. However, excessive temperature increases led to a decrease in the TPC since the extraction temperature exceeded the optimum X_1_, as in the case of over 50 °C, due to oxidative degradation and the decrease in solvent ability to dissolve the bioactive compounds, where more than half of the volume (50%) of the solvent was evaporated. In fact, the present findings are analogous to the results reported. For this reason, milder heating conditions are considered appropriate for the extraction, with the optimum condition taken at 40 °C. Figure 1b displays the interaction response surface plot of X_2_ and X_3_ and their interactions on the TPC. X_2_ is an important parameter in X_3_ because it affects the solubility and mass transfer of bioactive compounds. Furthermore, as shown in Figure 1b, the TPC increased with an increase in X_2_ up to 40 min, and then decreased slightly. Prolonged X_2_ increases the possibility of oxidation and epimerization, likely due to solvent saturation and the degradation of bioactive compounds. The optimum TPC is achieved with X_3_ of 30 mL/g and X_2_ of 30 min. These results explain the critical role of extraction time in minimizing extraction process costs. Figure 1c describes the interactive effects of X_1_ and X_3_ on the TPC, illustrating the interactions between X_1_ and X_3_ observed in the UAE of TPC. Results showed that when the X_3_ ratio increased from 1:20 to 1:30 g/mL, the solubility of *V. sinaiticum* also increased, leading to an increase in extraction efficiency. Moreover, the TPC extraction temperature was not significantly affected when X_1_ ranged from 30–50 °C; therefore, it does not affect the minimization or maximization of the TPC response. The effect of X_3_ on optimization was studied to increase the extraction efficiency, as well as decrease production cost and solvent usage.

UAE and ME methods were also employed for the extraction of total polyphenols from *V. sinaiticum*, as presented in Table 3. UAE emerged as the optimal method based on the desirability function of the TPC responses. The TPC of *V. sinaiticum* demonstrated significant differences (*p* < 0.05), while the lack of fit was not significant for these parameters. However, TPC showed significance (*p* < 0.001) in the model, indicating its impact. UAE, utilizing 70% ethanol, exhibited the highest TPC of 179.8 (±0.11) GAE mg/g in X_2_ of 30 min at X_1_ of 40 °C with X_3_ of 30 mL/g. This surpassed the TPC obtained through maceration methods (156.85 mg GAE/g) over 72 h with the same X_3_ of 30 mL/g. These values of ultrasonic and conventional extraction of TPC were higher than those in the previous studies of *V. sinaiticum* using methanol 80% extraction, which yielded 167 mg GAE/g [3]. These findings are consistent with similar studies reported on *M. stenopetala* extracts by Dadi D et al. [40]. The optimized UAE method simultaneously achieved the highest TPC of *V. sinaiticum* and antioxidant capacity within a short duration, while consuming less energy. The results show that the UAE method is more efficient than the ME method, as reported in similar studies [39].

The CCD of the optimization technique used to optimize the UAE process conditions and responses was considered [41]. The TPC was computed to determine the optimal conditions for the UAE procedure, with settings chosen to maximize the TPC, achieving desirability ratings of 1.000. The experimental and predicted values of the independent variables (X_1_, X_2_, and X_3_) and dependent parameter (TPC) were shown on Table 4. The optimum UAE parameters of X_1_ (41.42 °C), time (33.22 min) and X_3_ (36.32 mL/g) were with the predicted TPC value of 178.74 GAE/g dw. The experimental TPC value obtained through UAE was 179.8 mg GAE/g dw, which did not differ significantly from the predicted value.

### 3.3. Characterization of Phenolic Compounds Using UHPLC-ESI-QTOF-MS/MS

The type of solvent and extraction procedure are determinants of the extent of isolation of bioactive components from plants [8,9,10]. In this study, the optimized UAE method, with a solid-to-solvent ratio of 1:30 g/mL, extracted for 30 min at 40 °C with 70% ethanol, was used to obtain the sample extract of *V. sinaiticum* leaves. The secondary metabolites, phenolic compounds in particular, provide numerous benefits including their use in food components and pharmaceuticals. Analysis was made by UHPLC on an Agilent 1290 and MS 6545 series using a YMC-Pack Pro C18, 150 × 4.6 mm I.D. S-3 µm, 12 nm (Temp: 40 °C), and a gradient solvent system (A: 0.1% formic acid in DW; B: acetonitrile (ACN)) with a flow rate of 0.5 mL/min for 50 min. Identification of the potential secondary metabolites found in bioactive ultrasound-assisted hydro-ethanol (70%) extract of *V. sinaiticum* leaf was mainly made by UHPLC-ESI-QTOF-MS/MS.

The total ion chromatogram (TIC) data acquired were utilized for the tentative identification of metabolites in the extract. Both negative and positive modes of ionization were employed for their respective advantages in metabolite identification. Although the two modes revealed similar metabolites, the negative ion mode yielded a greater number of metabolites. Therefore, it was selected for the final identification of compounds in this study. Tentative identification of metabolites was accomplished by comparing spectral data with those available in reputable databases such as Metlin and Metabolomics Workbench (with less mass error and a library score of more than 80) and further validated through cross-referencing with relevant literature reports.

**Figure 2:**
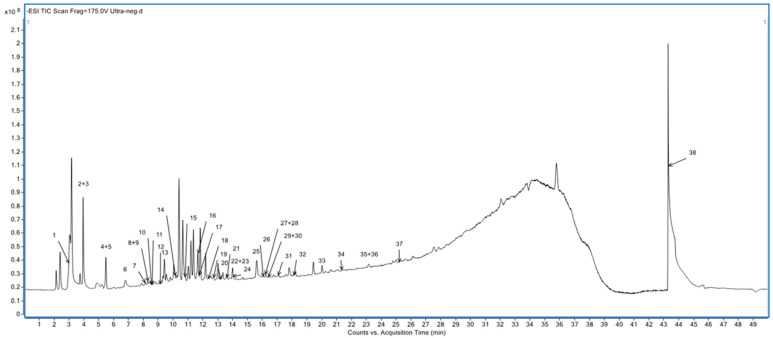
TIC of UHPLC-ESI-QTOF-MS/MS analysis extract of *V. sinaiticum* (negative mode).

The chromatographic patterns and mass spectral information of the ultrasound-assisted 70% ethanol extract of *V. sinaiticum* leaf led to 36 single-component tentative identifications of the mixture comprising carbohydrates/glycosides, iridoids, flavonoids, phenolics, fatty acids, oligopeptides, flavones, saponins, quinones, terpenoids, and alkaloids (Figure 2, Table 5). In the list, there are about 19 prospective metabolites responsible for antioxidant capacity; these are iridoids, quinones, flavonoids, and phenolic compounds.

The flavonoids identified in this analysis are quercetin 3-glucuronide-7-rutinoside ([M+HCOO]^−^, *m*/*z* 831.1854), hirsutin (M^+^; *m*/*z* 669.2026), miconioside A ([M+HCOO]^−^, *m*/*z* 653.2081), saponarin ([M-H]^−^; *m*/*z* 593.1506), isoaffinetin ([M-H]^−^; *m*/*z* 463.0873), diosmin ([M-H]^−^; *m*/*z* 607.1665), quercetin 5,7,3′,4′-tetramethyl ether 3-rutinoside ([M-H]^−^; *m*/*z* 665.2074), catechin 7,4′-di-O-gallate ([M-H]^−^; *m*/*z* 593.0923), 4′-hydroxy-5,7,2′-trimethoxyflavanone 4′-rhamnosyl-(1->6)-glucoside ([M+CH_3_COO]^−^; *m*/*z* 697.2334), luteolin 7-O-(2-apiofuranosyl-4-glucopyranosyl-6-malonyl)glucopyranoside ([M-H]^−^; *m*/*z* 827.1898), 4′-Hydroxy-5,7,2′-trimethoxyflavanone 4′-rhamnosyl-(1->6)-glucoside ([M+CH_3_COO]^−^;*m*/*z* 697.2342), luteolin ([M-H]^−^; *m*/*z* 285.0404), naringenin 7-O-(2″,6″-di-O-alpha-rhamnopyranosyl)-beta-glucopyranoside ([M-H]^−^; *m*/*z* 725.2279), mopachalcone ([M-H]^−^; *m*/*z* 299.0554).

The phenolics, namely hellicoside ([M-H]^−^; *m*/*z* 655.1881), zingerone ([M-H]^−^; *m*/*z* 193.0867), and 8-caffeoyl-3,4-dihydro-5,7-dihydroxy-4-phenylcoumarin ([M+HCOO]^−^, *m*/*z* 463.1031), were detected from the plant extract using UHPLC-ESI-QTOF-MS/MS (Table 5). The identification of these flavonoids/phenolic compounds in the plant extract confirmed its antioxidant capacity.

### 3.4. FTIR

**Figure 3:**
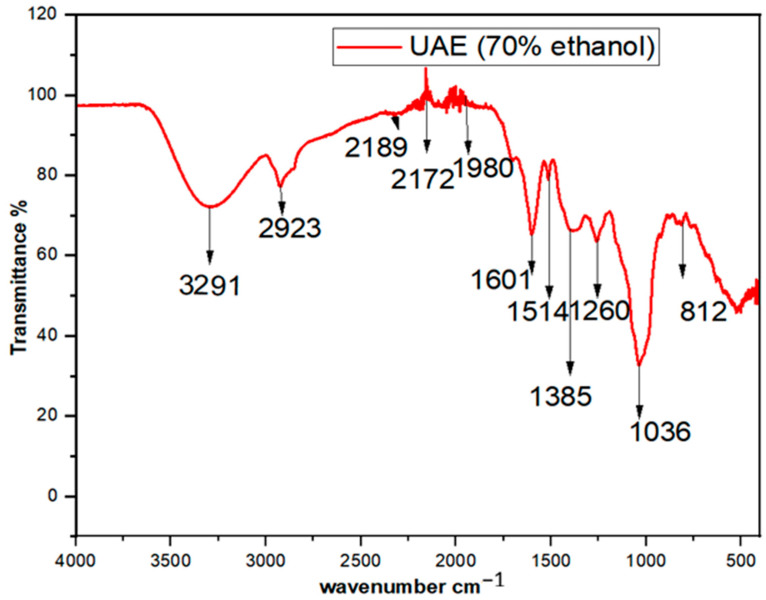
FTIR spectrum of dried ultrasound-assisted ethanol (70%) extract of shade-dried *V. sinaiticum* leaf.

The extract was analyzed by an FTIR spectrometer, and spectra were recorded within the 400–4000 cm^−1^ scanning range. The FTIR spectrum (Figure 3) showed absorptions assigned to phenolic O-H (3291), aromatic C-H (2923), C=C (1601), C≡N or C≡C or aromatic C-H bending (2050), C-O bending (1036), and phenolic O-H bending (1385). Generally, the detection of these functional groups supports the presence of phenolic metabolites, as reported by another researcher [13].

### 3.5. XRD

**Figure 4:**
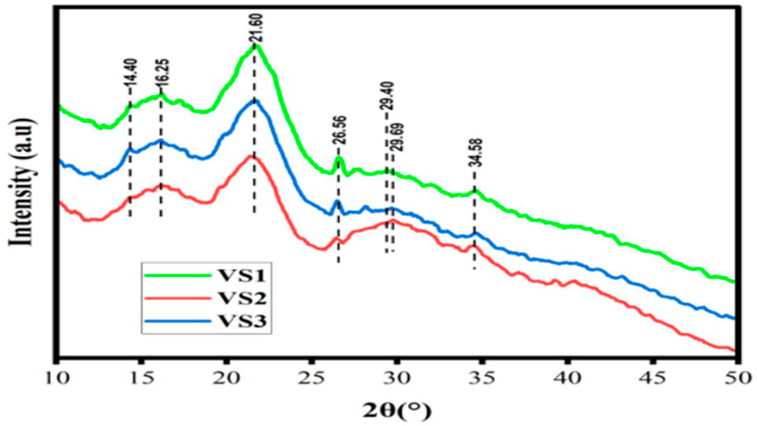
XRD curves of *V. sinaiticum* extracts: (VS_1_: Shade dryer; VS_2_: Fluidized bed dryer 70 °C; VS_3_: Oven dryer@105 °C), (2θ = 14.4°, 16.25°, 21.6°, 26.56°, and 34.58).

The crystallinity and structure of the dried and ultrasound-assisted extracted *V. sinaiticum* leaves powder using 70% ethanol were analyzed (Figure 4).

**Figure 5:**
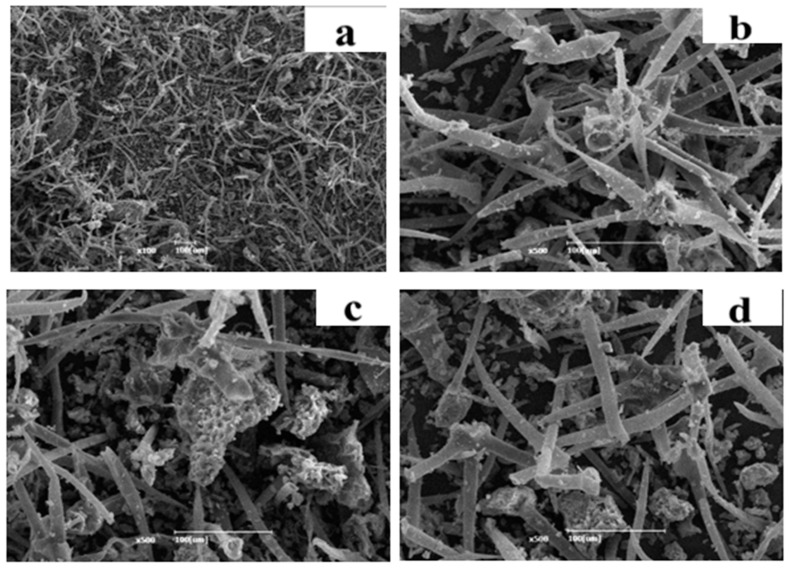
SEM image of the shade- and oven-dried *V. sinaiticum* leaf: (**a**) Before extraction; (**b**) shade dried after ethanol (70%) extraction); (**c**) shade-dried after UAE ethanol (70%) extraction, and (**d**) oven-dried after ethanol (70%) extraction.

All of them are broad and showed the presence of amorphous material in the sample. The diffraction peak around 21.6° is very intense and corresponds to the dominant component of the samples. Peaks at 2θ = 14.4°, 16.25°, 21.6°, 26.56° and 34.58° were observed in the XRD spectra. The XRD spectra of samples extracted by ultrasound-assisted ethanol (70%) (VS1), oven-dried ethanol (70%) extracted (VS2), and shade-dried ethanol extracted (70%) (VS3) exhibited identical patterns (Figure 5). The diffraction angle around 2θ = 21.6° indicates the presence of phenolic compounds in the dried matrix of the samples. The presence of phenolic compounds as the principal component in the extracts confirms the observed antioxidant potential of the plant extract [38,42].

In general, the plant, *V. sinaiticum* leaf, is rich in phytochemicals, and the presence of these bioactive constituents supports the traditional claim of the medicinal plant. Reportedly, compounds isolated from *V. sinaiticum* displayed dose-dependent cytotoxicity against leukemia cells [4,5,6]. The ethanolic extract of *V. sinaiticum* leaf is also reported to possess broad-spectrum antibacterial activity [7].

Therefore, assessing the potential radical scavenging capacity of the crude extract from the *V. sinaiticum* leaves containing the aforementioned polar compounds was the focus of this study.

### 3.6. SEM

Morphological analysis of the *V. sinaiticum* extract residue was performed (Figure 5). This analysis revealed that there were minor variations corresponding to the type of extract methods (ME and UAE). The smooth appearance of the images of the residues after extraction is due to the tight-linked micro-fibrils with adhesive amorphous components, waxes, and oils of similar morphology. The higher pores size observed by the ultrasound-assisted extracted residues (c) relative to that of the no extracted powder. This showed the presence of a high degree of isolation of secondary metabolites.

## 4. Conclusions

This study investigated the effects of three factors, namely temperature (20–40 °C), extraction time (20–40 min), and solvent-to-solute ratio (30–50 mL/g), using CCD in ultrasound-assisted extraction. UAE proved superior to conventional methods, providing a higher quality and yield of extracts from *V. sinaiticum* leaves in a shorter time. The highest extraction yields (21.60%), TPC of (179.8 mg GAE/g), and TFC of (64.49 mg CE/g) were achieved at an extraction temperature of 40 °C, a time of 30 min, and a solvent-to-solute ratio of 30 mL/g. Additionally, the highest antioxidant values with ABTS (61.85 IC50 µg/mL) and DPPH (38.89 IC50 µg/mL) assays were recorded under these parameters. A CCD was employed to optimize TPC values, with ANOVA analysis revealing significant quadratic polynomial models. The linear term of the solvent-to-solute ratio (X_3_) and the quadratic term of extraction time (X_2_^2^) had the largest effects on TPC. The optimum UAE conditions were determined as 40 min of sonication time (X_2_), 40 °C extraction temperature (X_1_), and a solute-to-solvent (X_3_) ratio of 1:30 g/mL. The experimental TPC value was closely matched to the predicted value, validating the optimization process, while the predicted TPC values were 178.74 GAE mg/g dw at 41.42 °C, 33.22 min, and 36.32 mL/g. UHPLC-QTOF MS/MS analysis of the extract revealed the presence of various bioactive compounds, including carbohydrates, iridoids, flavonoids, and phenolics, suggesting its potential for pharmaceutical and food applications. The phytochemical composition of the extract indicates promising prospects for the development of novel products in the food and pharmaceutical industries.

## Figures and Tables

**Table 1:** Levels of independent variables for experimental design.

Symbols	Independent Variables	Factor Level		
		−1	0	+1
X_1_	Temperature (°C)	30	40	50
X_2_	Time (min)	20	30	40
X_3_	Solvent-to-solute ratio (mL/g)	20	30	40

**Table 2:** Experimental design and levels of *V. sinaiticum* process variables CCD.

Run	X_1_ (°C)	X_2_ (Min)	X_3_ (mL/g)	Yield (%)	TPC (mg GAE/g)	TFC (mg CE/g)	DPPH IC50 (µg/mL)	ABTS IC50 (µg/mL)
1	30	20	20	19.97	156.69	37.55	45.88	28.24
2	50	20	20	20.33	157.14	38.06	45.93	29.45
3	30	40	20	20.48	159.8	41.55	46.5	29.48
4	50	40	20	20.77	169.28	51.1	47.13	29.45
5	30	20	40	20.5	164.92	44.61	46.8	29.26
6	50	20	40	20.57	165.71	46.27	46.88	29.94
7	30	40	40	20.65	167.78	50.78	46.98	27.99
8	50	40	40	20.84	169.31	51.31	47.23	28.76
9	23.2	30	30	20.69	168.7	50.99	47.1	29.48
10	56.8	30	30	20.89	169.88	51.41	49.52	30.71
11	40	13.2	30	19.35	151.03	32.45	44.23	28.88
12	40	46.8	30	21.58	171.78	61.55	56.88	35.24
13	40	30	13.2	19.95	156.03	35.67	44.93	28.62
14	40	30	46.8	21.59	179.09	59.47	57.08	35.42
15	40	30	30	21.6	179.8	64.49	52.45	35.88
16	40	30	30	21.53	178.61	58.73	56.63	32.57
17	40	30	30	21.53	177.99	53.98	54.3	32.31
18	40	30	30	21.53	175.65	53.57	54.1	30.1
19	40	30	30	21.5	174.7	52.96	61.85	38.89
20	40	30	30	21.15	172.76	51.41	49.67	29.49
	**ME**	72 h	30 mL/g	20.85 ± 0.2	156.85 ± 0.09	34.14 ± 0.04	52.15 ± 0.01	31.34 ± 0.05

Yield: %; TFC: total flavonoid content; CE: catechin equivalent; TPC: total phenolic content; gallic acid equivalent; dry weight of the sample; DPPH and ABTS; IC50: inhibition 50%; X_1_: extraction temperature; X_2_: time; X_3_: solvent-to-solute ratio, ME: maceration extraction.

**Table 3:** Analysis of variance and regression coefficients for TPC response variable.

Independent Variable	Dependent Variable (Response)				
Factors	Yield (%)	TPC (mg GAE/g)	TFC (mg CE/g)	DPPH IC50 (µg/mL)	ABTS IC50 (µg/mL)
Intercept	21.46	176.51	80.37	51.79	41.01
Linear					
X_1_-temperature	0.0755	1.03	+2.99	+0.0907	+0.0907
X_2_-sonication time	0.9688	4.15	+9.11	+0.3745	+0.3745
X_3_-solvent-to-solute ratio	0.5943	4.74	+4.76	+0.2769	+0.2769
Interaction					
X_1_∗X_2_	0.0375	1.22	−2.69	+0.0063	+0.0063
X_2_∗X_3_	−0.0125	−0.9500	−4.18	−0.0487	−0.0487
X_1_∗X_3_	−0.6375	−1.10	−3.25	−0.0662	−0.0663
quadratic					
X_1_^2^	−0.2847	−2.88	−1.77	−0.2629	−0.2629
X_2_^2^	−0.7864	−5.62	−6.97	−0.3753	−0.3753
X_3_^2^	−0.3020	−3.37	−1.67	−0.2681	−0.2681

X_1_: temperature; X_2_: sonication time; X_3_: solvent-to-solute ratio; X_1_∗X_2_: temperature and sonication time; X_1_∗X_3_: temperature and solvent-to-solute ratio; X_2_∗X_3_: solvent-to-solute ratio and time; X_1_^2^: temperature∗temperature; X_2_^2^: time∗time; X_3_^2^: solvent-to-solute ratio∗solvent-to-solute ratio.

**Table 4:** Optimized UAE parameters and TPC.

	Temperature (°C)	Solvent-to-Solute Ratio (mL/g)	Time (min)
Optimized parameters	41.4261	36.3171	33.2215
Predicted values	178.741 mg GAE/g dw		
Experimental value	179.800 mg GAE/g dw		

**Table 5:** List of metabolites identified by UHPLC-ESI-QTOF-MS/MS (negative mode) analysis of the UAE of *V. sinaiticum*.

Peak	RT/min	[M-H]^−^ and Other (*m*/*z*)	Diff (DB, mDa)	Molecular Weight	Formula	Identified Compound Name
1	2.992	827.267	−0.61	828.2742	C_30_H_52_O_26_	Verbascose
2	3.958	393.1395	−1.56	348.1415	C_15_H_24_O_9_	Leonuridine
3	3.959	290.088	−0.31	291.0953	C_11_H_17_NO_8_	Sarmentosin epoxide
4	5.487	373.1138	−0.75	374.121	C_16_H_22_O_10_	Gardoside *
5	5.488	831.1854	2.3%	786.1855	C_33_H_38_O_22_	Quercetin 3-glucuronide-7-rutinoside ***
6	6.812	101.0604	−4.16	102.0677	C_5_H_10_O_2_	Pivalic acid
7	8.141	475.1814	−1.29	476.1888	C_21_H_32_O_12_	Kanokoside A
8	8.3	669.2031	0.05	669.2026	C_30_H_37_O_17_	Hirsutin ***
9	8.317	785.2497	−2.04	786.2582	C_35_H_46_O_20_	Magnoloside B
10	8.551	403.1607	0.66	344.1471	C_16_H_24_O_8_	Iridotrial glucoside *
11	8.582	435.1497	−1.06	390.1515	C_17_H_26_O_10_	Loganin *
12	9.146	655.1881	−0.2	656.1953	C_29_H_36_O_17_	Hellicoside ****
13	9.416	653.2081	−1.05	608.2099	C_29_H_36_O_14_	Miconioside A ***
14	10.161	593.1506	−1.03	594.1579	C_27_H_30_O_15_	Saponarin ***
15	10.81	463.0873	−2.07	464.0945	C_21_H_20_O_12_	Isoaffinetin ***
16	11.665	608.1736	−0.79	607.1665	C_28_H_32_O_15_	Diosmin ***
17	11.77	665.2074	−0.66	666.216	C_31_H_38_O_16_	Quercetin 5,7,3′,4′-tetramethyl ether 3-rutinoside ***
18	12.409	593.0923	−2.06	594.101	C_29_H_22_O_14_	Catechin 7,4′-di-O-gallate ***
19	12.75	401.1445	−2.37	342.1307	C_16_H_22_O_8_	Coniferin
20	13.217	697.2334	−2.23	638.2211	C_30_H_38_O_15_	4′-Hydroxy-5,7,2′-trimethoxyflavanone 4′-rhamnosyl-(1->6)-glucoside ***
21	13.649	354.2394	−0.5	309.2411	C_17_H_31_N_3_O_2_	Palustrine
22	13.998	827.1898	−2.03	828.1974	C_35_H_40_O_23_	Luteolin 7-O-(2-apiofuranosyl-4-glucopyranosyl-6-malonyl)glucopyranoside ***
23	14.00	697.2342	1.6	638.2201	C_30_H_38_O_15_	4′-Hydroxy-5,7,2′-trimethoxyflavanone 4′-rhamnosyl-(1->6)-glucoside ***
24	14.051	841.457	−2.44	796.4609	C_42_H_68_O_14_	Soyasaponin III
25	15.625	285.0404	−0.11	286.0477	C_15_H_10_O_6_	Luteolin ***
26	16.052	987.5151	−2.04	942.5169	C_48_H_78_O_18_	Soyasaponin I
27	16.227	725.2279	−2.27	726.2371	C_33_H_42_O_18_	Naringenin 7-O-(2″,6″-di-O-alpha-rhamnopyranosyl)-beta-glucopyranoside ***
28	16.231	755.2395	−1.66	756.2477	C_34_H_44_O_19_	Myricoside
29	16.415	463.1031	−1.16	418.1053	C_24_H_18_O_7_	8-Caffeoyl-3,4-dihydro-5,7-dihydroxy-4-phenylcoumarin ****
30	16.417	369.1183	−2.53	310.1053	C_15_H_18_O_7_	Mellitoxin
31	17.095	327.2172	−1.67	328.2244	C_18_H_32_O_5_	9-hydroperoxy-12,13-epoxy-10-octadecenoic acid
32	18.125	299.0554	−0.67	300.0627	C_16_H_12_O_6_	Mopachalcone ***
33	20.00	433.092	−1.80	434.0994	C_24_H_18_O_8_	Knipholone **
34	21.371	193.0867	−1.65	194.0943	C_11_H_14_O_3_	Zingerone ****
35	23.15	221.1539	−3.38	222.162	C_14_H_22_O_2_	Rishitin
36	23.151	293.1754	−1.69	294.1831	C_17_H_26_O_4_	Embelin **

*: iridoids, **: quinones, ***: flavonoids, and ****: phenolic.

## Data Availability

The original contributions presented in the study are included in the article, further inquiries can be directed to the corresponding author.

## Abbreviation

ABTS	2-azino-bis 3-ethylbenzeothiazoline-6-sulfonic acid diammonium salt
ACN	acetonitrile
CE	catechin Equivalent
CCD	central composite design
DPPH	2,2,-diphenyl-1-picrylhydrazyl
Equ	equation
FBD	fluidized bed dryer,
FTIR	Fourier transform infrared
GAE	gallic acid equivalent
h	hour
ME	maceration
Mg CE/g D	milligram cathechin equivalent per gram dry extract
Min	minutes
Ov	oven dryer
SEM	scanning electron microscopy
TFC	total flavonoid content
TIC	total ionic component
TPC	total phenolic content
UAE	ultrasound-assisted extraction
UPHLC-QTOF MS/MS	ultra-high-performance liquid chromatography quadrupole time of flight mass spectroscopy
*V. sinaiticum*	*Verbascum sinaiticum*
XRD	X-ray diffractometer
